# Lack of genetic differentiation in yellowfin tuna has conservation implications in the Eastern Pacific Ocean

**DOI:** 10.1371/journal.pone.0272713

**Published:** 2022-08-30

**Authors:** Laia Muñoz-Abril, Maria de Lourdes Torres, Carlos A. Valle, Francisco Rubianes-Landázuri, Felipe Galván-Magaña, Steven W. J. Canty, Martin A. Terán, Margarita Brandt, Jaime A. Chaves, Peter M. Grewe

**Affiliations:** 1 Colegio de Ciencias Biológicas y Ambientales COCIBA, Universidad San Francisco de Quito USFQ, Diego de Robles y Pampite, Quito, Ecuador; 2 Department of Marine Sciences, University of South Alabama, USA Drive North, Mobile, Alabama, United States of America; 3 Instituto Politécnico Nacional, Centro Interdisciplinario de Ciencias Marinas, La Paz, México; 4 Smithsonian Marine Station Fort Pierce, Fort Pierce, Florida, United States of America; 5 Working Land and Seascapes, Smithsonian Institution, Washington, DC, United States of America; 6 Department of Biology, San Francisco State University, San Francisco, CA, United States of America; 7 CSIRO Oceans & Atmosphere, Castray Esplanade, Hobart, Tasmania, Australia; University of Iceland, ICELAND

## Abstract

Yellowfin tuna, *Thunnus albacares*, is an important global fishery and of particular importance in the Eastern Pacific Ocean (EPO). According to the 2019 Inter-American Tropical Tuna Commission (IATTC) assessment, yellowfin tuna within the EPO is a single stock, and is being managed as one stock. However, previous studies indicate site fidelity, or limited home ranges, of yellowfin tuna which suggests the potential for multiple yellowfin tuna stocks within the EPO, which was supported by a population genetic study using microsatellites. If numerous stocks are present, management at the wrong spatial scales could cause the loss of minor yellowfin tuna populations in the EPO. In this study we used double digestion RADseq to assess the genetic structure of yellowfin tuna in the EPO. A total of 164 yellowfin tuna from Cabo San Lucas, México, and the Galápagos Islands and Santa Elena, Ecuador, were analysed using 18,011 single nucleotide polymorphisms. Limited genetic differentiation (F_*ST*_ = 0.00058–0.00328) observed among the sampling locations (México, Ecuador, Peru, and within Ecuador) is consistent with presence of a single yellowfin tuna population within the EPO. Our findings are consistent with the IATTC assessment and provide further evidence of the need for transboundary cooperation for the successful management of this important fishery throughout the EPO.

## Introduction

Overfishing is one of the greatest threats to the yellowfin tuna, *Thunnus albacares* [1]. The International Union for the Conservation of Nature (IUCN) has categorized this species as least concern, with a declining population trend [2]. Five intergovernmental associations, the Tuna Regional Fisheries Management Organizations (tRFMOs), are dedicated to monitor and manage tuna and tuna-like populations [3]. Some of these organizations have worked on rebuilding stocks, most noticeably those of Atlantic bluefin tunas [4]. Four tRFMOs manage populations of the yellowfin tuna, these are: the International Commission for the Conservation of Atlantic Tunas (ICCAT); the Indian Ocean Tuna Commission (IOTC); the Western and Central Pacific Fisheries Commission (WCPFC); and the Inter-American Tropical Tuna Commission (IATTC) [5]. Efforts to rebuild yellowfin tuna fisheries have been implemented throughout the species range [3], which has included reducing quotas of fleets that surpassed their limits [6].

Various studies suggest that, at a global level, yellowfin tuna is split into several populations. Genetic assessments by tRFMOs have reported the presence of four different stocks, which are located in the Atlantic Ocean, Indian Ocean, Eastern Pacific Ocean (EPO), and West Central Pacific Ocean [5, 7–9]. However, the finer resolution of population structure within oceans shows contradictory results, which could be partially attributable to the lack of a suitable model for the analysis of genetic marker data of marine species. The latest assessment on the status of yellowfin tuna in the EPO assume the existence of a single stock [10]. However, Díaz-Jaimes & Uribe-Alcocer [11] suggest genetic differentiation between yellowfin tuna sampled in the northern (Gulf of California, Mexican coast and southwest Revillagigedo Islands) and southern EPO (Perú). Their study used microsatellite markers (7 loci) to detect genetic differences among subregions; a method that is not always reliable for determining the genetic structure in species like tunas due to their large effective population sizes [12]. Ely et al. [13] estimated the long term effective population size of females with mismatched distributions of D-loop sequences, suggesting a large long-term number of effective female breeders and low levels of genetic differentiation between Atlantic and Pacific yellowfin tuna individuals. Conversely, Barth et al. [8] estimated the demographic history of yellowfin tuna, detecting significant differentiation of Atlantic and Indo-Pacific yellowfin tuna populations and showing that the true values of effective size (Ne) are far below those estimated by Ely [13], which may suggest greater genetic differentiation within populations. However, the sample size used was smaller than 12 individuals per sampling area. Estimations such as the one for Atlantic bluefin tuna with large sample size have not been carried out in yellowfin tuna [14].

In addition to genomic analyses, a number of other studies suggest that yellowfin tuna have a home range of 1,800 km, and exhibit high regional fidelity [15–21]. Studies which support the high fidelity theory include, analyses of yellowfin tuna parasites [22], stable isotopes [23], and pop-up satellite tags [24, 25]. The findings of these studies support what Díaz-Jaimes & Uribe-Alcocer found [11], however, as we previously mentioned, the use of microsatellite in their analyses could have some limitations due to their sample size and the utility of microsatellites. The limitations of microsatellites can be overcome using Next Generation Sequencing (NGS); a meta-analysis of yellowfin tuna population genetic studies emphasised the importance of NGS over other techniques [26]. NGS techniques offer a broader representation of the genome and allows the identification of single nucleotide polymorphisms (SNPs) loci that are possibly undergoing selection. SNPs can be found in DNA fragments less than 100 bp in size versus microsatellites that have larger amplicons [27]. Though more markers are required for SNPs analyses, high numbers of SNPs provide greater power for assessing genetic structure [28]. Additionally, these loci provide a better opportunity to distinguish populations with low differentiation, by discriminating neutral evolutionary processes like genetic drift from those caused by selection that could result in local adaptations [29].

Hauser and Carvalho [30] emphasize the need for regular updates of population studies of commercial fish species because populations vary over time, and there is a risk of losing minor populations when multiple populations are managed as a single stock [31]. Considering that several studies suggest residence and limited movement of yellowfin tuna, as well as structured populations (albeit with a small sample size), multiple yellowfin tuna stocks may be present in the EPO. Currently, yellowfin tuna in the EPO is managed as a single stock, which could cause the loss of minor yellowfin tuna populations in the region, if they exist. NGS approaches in marine organisms provide a finer resolution of population structure using several thousand SNPs [32, 33], and is suggested to be the most appropriate tool to yellowfin tuna populations [26]. Integrating genomic data into fisheries management is a critical step to improve management practices, to ensure the ecological sustainability of fish stocks [34]. By using NGS approaches, this study tested the hypothesis that a single genetic stock of yellowfin tuna exists within the EPO, to determine the most suitable spatial scale for the effective management of this important fishery.

## Materials and methods

### Sample collection

**Fig 1:**
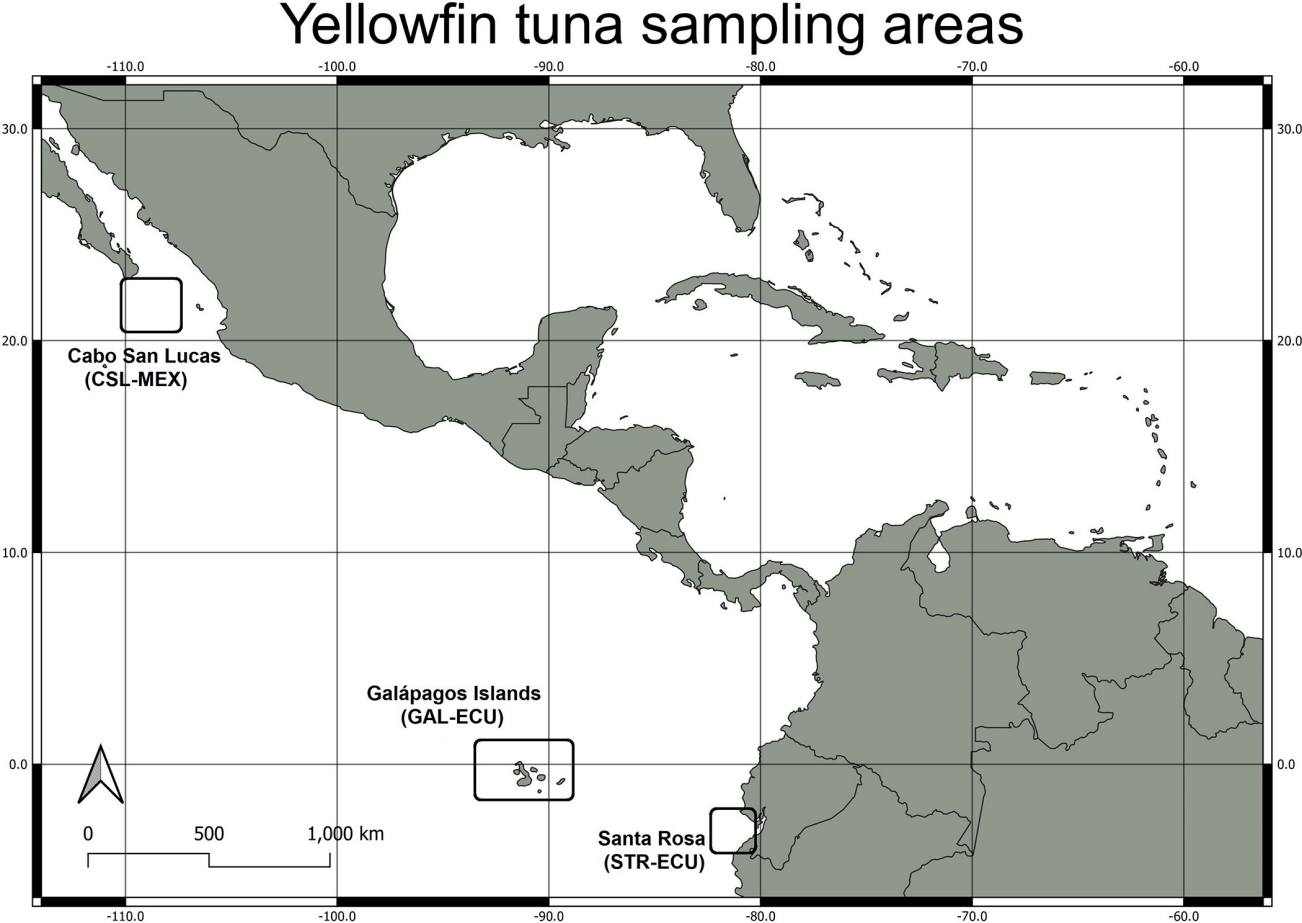
Approximate sampling areas of yellowfin tuna in the Eastern Pacific Ocean. This figure was produced in QGIS.

Yellowfin tuna samples were collected from three artisanal fishing ports each associated with distinct fishing grounds: [1] **CSL-MEX**: Cabo San Lucas (20.985°N, -109.539°W), La Paz, México, covering the northern tropical EPO; [2] **GAL-ECU**: Santa Cruz (0.132°S, -91.383°W), Galápagos Islands, Ecuador, covering the central tropical EPO; and [3] **STR-ECU**: Santa Rosa fishing port (-4.022°S, -82.953°W), Santa Elena, Ecuador, covering the continental coast of Ecuador and northern Perú (Fig 1). Sampling occurred over a three-year period, from February 2015 to October 2017, under permits MAE-DNB-CM-0046 and MAE-DNB-CM-2016-0041. All yellowfin tuna sampled in this study were caught by artisanal fishing fleets for commercial purposes and no fish was euthanized for this research. Samples from a total of 230 individual yellowfin tuna were initially collected, 64 from CSL-MEX, 103 from GAL-ECU, and 63 from STR-ECU. The sample size of this study is comparable with other studies (20–40 samples per location) testing similar hypotheses [7, 14, 35] and enough to provide an accurate genetic assessment of the yellowfin tuna population [36].

Fork length (FL) of each individual fish was measured to the nearest centimetre (cm) using a fish board; fish ranged in size from 34–200 cm FL. A sample of red muscle, approximately 1cm^3^ in size, was removed from individual yellowfin tunas, at fishing landing sites. New disposable scalpels were used to collect each sample, to avoid cross contamination. Muscle samples were stored in individually labelled Eppendorf tubes in 97% ethanol (ensuring the entire sample was immersed) for transportation and further processing. Sexing individuals requires the extraction of their gonads, which in turn reduces the commercial value of the fish. Consequently, we could not determine our samples’ sexes as fish were sampled on the proviso that they retained their market value.

### ddRAD genotyping and quality filtering

DNA extraction, double digestion RAD library preparation and sequencing, and SNP genotyping were carried out by Diversity Arrays Technology (DArT, Canberra, Australia). The patented protocol DArTseq™ was used as it is a cost-effective methodology, and has been optimized for the analysis of yellowfin tuna high-throughput genotyping data [7, 37, 38]. Following automated DNA extraction using Tecan Freedom EVO 150 robots (Männedorf, ZH, Switzerland), samples were digested using *PstI* and *SphI* methylation-sensitive restriction enzymes. These enzymes were chosen as they avoid the repetitive fractions of the genome, which is uninformative for SNP discovery [26, 38], and also because they have been used in previous NGS studies of yellowfin tuna [7]. *PstI* and *SphI* compatible adapters, which included an Illumina flow cell attachment sequence and a PCR primer sequence, were ligated to the restriction fragments. Additionally, the *PstI* adapter incorporated unique barcode sequences to identify samples within pooled libraries. Only fragments capped with both *PstI* and *SphI* adaptors were amplified. PCR conditions consisted of an initial denaturation step at 94°C for 1 min, followed by 30 cycles at 94°C for 20 sec, 58°C for 30 sec, and 72°C for 45 sec, with a final extension step at 72°C for 7 min. Bridge PCR was then applied to further amplify and sequence the libraries on the Illumina HiSeq 2500 (San Diego, CA, USA) using MJ Tetrad PCR thermocyclers (Hercules, CA, USA) [7].

The resulting sequences were processed using DarTseq analytical pipelines DarT-Soft14 version [7]. The primary quality control pipeline consisted of two filtering steps of the FASTQ files, applying decreasingly stringent selection criteria to the barcode region to increase demultiplexing accuracy. The first step stringently filters out barcode sequences with a Phred score threshold of 30, and the second step filters out sequencing reads with Phred score threshold of 10 [39]. Identical sequences were then collapsed into fastqcoll files and “groomed” using DarT’s proprietary algorithm. This algorithm replaces a low-quality base from a singleton tag with the correct base through a template composed of collapsed tags with multiple members. The corrected fastqcoll files were then used in the DArT-Soft14 secondary pipeline for SNP calling. The final dataset produced by these pipelines consisted of individual genotype reports for each processed sample presented as a matrix of SNP loci, their global call rate, polymorphic information content (PIC), and their co-dominant status.

The SNP dataset was further filtered using the package radiator [40] in R v3.5.3 [41] to remove outliers and noise that could interfere with polymorphism estimates. Filter parameters for SNP quality control included DArT reproducibility (proportion of reproducible genotypes calculated through the comparison of technical replicates), monomorphic markers (to remove markers in one unique genotype), common markers (to remove markers not found across all individuals), minor allele count, call rate (proportion of samples successfully genotyped), SNP number (maximum number of SNPs per marker), SNP position on the fragment sequenced, linkage disequilibrium, Hardy-Weinberg Equilibrium, duplicated genomes (potential duplicated samples estimated via pairwise genome similarity) and mixed genomes (potential mixed samples or poor SNP discovery caused by DNA quality, sequencing procedure, among others). Quality control for individual samples with low DNA quality or DNA contamination included filters of missingness (proportion of non-genotyped values), heterozygosity (proportion of heterozygous loci) and total coverage (average sequencing depth across markers) (S1 Table). DArT DS14 pipeline delivered 80,000 filtered markers. After filtering using radiator [40], a total of 164 individuals: 35 from CSL-MEX, 88 from GAL-ECU, and 41 from STR-ECU; and 18,011 loci were retained after quality control (S1 Table) and used for the remaining analyses.

### Data analysis

Basic genetic diversity measures of the quality controlled data set were conducted in R v3.5.3 [41]. Population genetic statistics such as Allelic richness (Ra), observed heterozygosity (Ho) and expected heterozygosity (He) were calculated using the adegenet package [42]. A Mann Whitney test was applied to determine the statistical significance of observed heterozygosity. Analyses for Molecular Variance (AMOVA) were run in the R package Poppr [43]. Global and pairwise F_*ST*_ values were calculated using DartR [44], and the inbreeding coefficient was calculated using the R package InbreedR [45]. The mean probability of identity by descent across loci was calculated using the R package rrblup [46]. Population genetic structure among the three study areas was assessed by genetic differentiation (F_*st*_), the absolute allele frequency difference (AFD) [47], Discriminant Analysis of Principal Components (DAPC), Principal Component Analysis (PCA), and non-spatial clustering (Structure). To determine if any observed population structuring was a result of isolation by distance, a Mantel test was performed. All analyses were conducted using the R package DartR [44]. PCA and non-spatial clustering analysis were conducted in STRUCTURE [48] and CLUMPP [49] software packages using the StrataG interface [50]. STRUCTURE was run with and without admixture with prior information on geographic origin included. Run parameters including the range of K (1–10) values assessed, the number of replicates, as well as burning and number of iterations were set on default values. The outputs produced by STRUCTURE were introduced in the Structure Selector [51] and Clumpak [52] to evaluate the optimal value of K, which was K = 1. According to Li & Liu, the Evanno method could underestimate the results [51]. Structure Selector [51] uses four statistical methods (MEDMEDK, MEDMEAK, MAXMEDK and MAXMEAK) to calculate the K number more accurately. A DAPC and PCA were generated in the adegenet package [42]. The DAPC was run without *a priori* grouping of samples through alpha optimization (via the dapc() and a.score.optim() commands).

## Results

### Genetic diversity

The global expected heterozygosity (Hs) was 0.14 and the overall observed heterozygosity (Ho) was 0.13 (S1 Fig). No statistical differences among the heterozygosity values (p = 0.493), discarding excess or deficit of heterozygotes were observed. The allelic richness was similar across all three sampling areas, 1.77 for CSL-MEX, 1.78 for GAL-ECU, and 1.75 for STR-ECU (Table 1). The AMOVA results show that the greatest variability occurs *within* individuals, with a mean value of 96.3%. Variation *among* individuals within sampling areas was 3.6%, while only 0.1% of the variation was explained by differences among sampling areas (S2 Fig).

**Table 1:** Summary of genetic diversity statistics for each sampling area of yellowfin tuna.

Locations	Ar	Ho	Hs
**CSL-MEX**	1.77	0.11 (0.02)	0.11
**GAL-ECU**	1.78	0.11 (0.01)	0.15
**STR-ECU**	1.75	0.10 (0.01)	0.10
**Total**		0.11 (0.02)	0.11

Ar: allelic richness, Ho: observed heterozygosity, Hs: expected heterozygosity. Standard deviation in parentheses.

### Genetic structure

**Fig 2:**
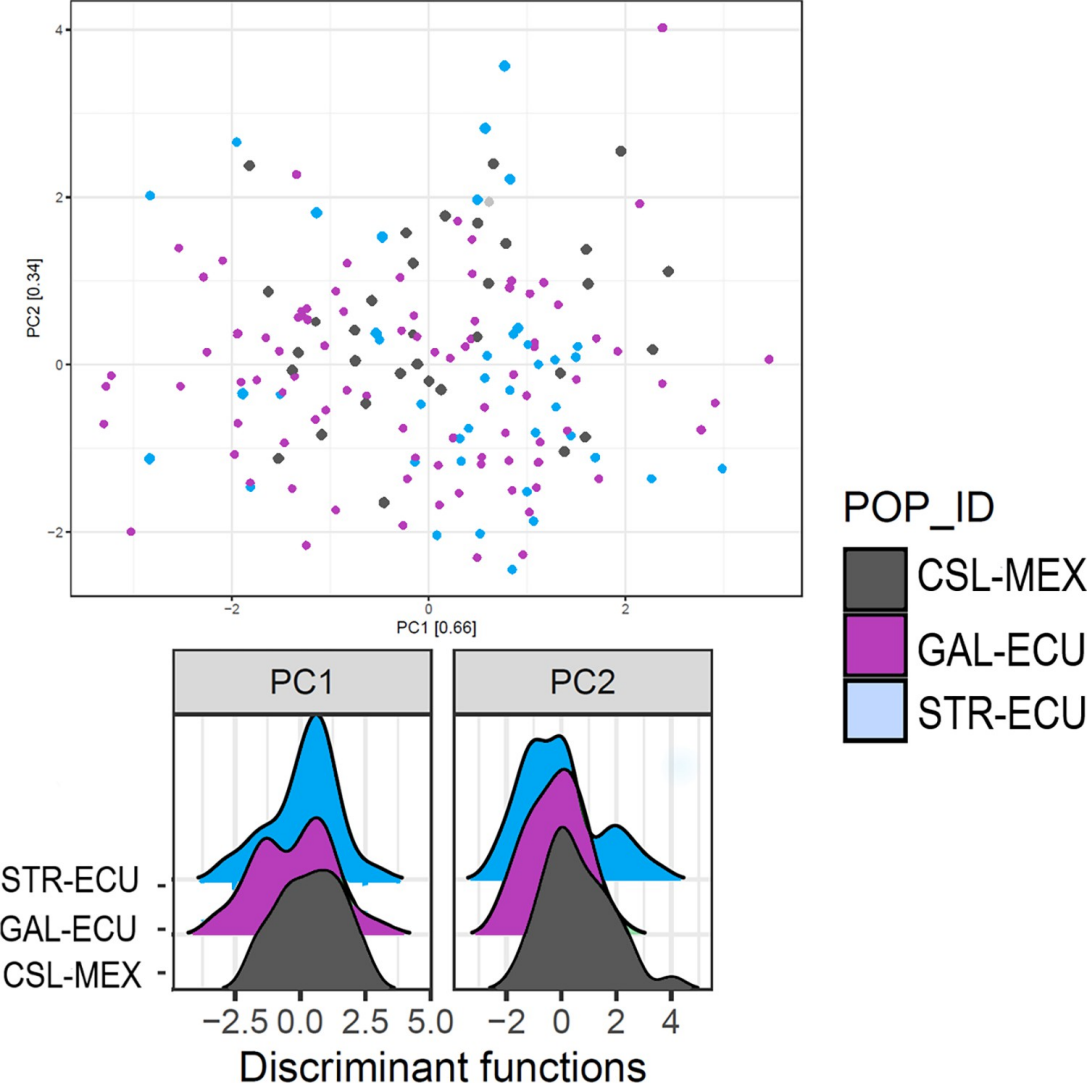
(A) Genetic clusters of yellowfin tuna using analysis of Principal Components (PC) identified by Adegenet (B) Discriminant Analysis of Principal Components of yellowfin tuna within the Eastern Pacific Ocean, using 18,011 markers grouping samples by sampling site. CSL-MEX: Cabo San Lucas, México (n = 35), GAL-ECU: Galápagos Islands (n = 88), Ecuador, STR-ECU: Santa Elena, Ecuador (n = 41).

Limited genetic differentiation (F_*ST*_ = 0.00328) was observed across the 164 individuals. Pairwise F_*ST*_ analyses identified similarly low genetic differentiation between the three sampling areas, with the greatest differentiation observed between CSL-MEX and STR-ECU (F_ST_ = 0.0005 followed by CSL-MEX and GAL-ECU (F_*ST*_ = 0.0004) and the lowest between GAL-ECU and STR-ECU (F_*ST*_ = 0.0003) (Table 2). No isolation by distance was observed (Mantel statistic r: 0.9452, p = 0.16). Low allele frequency difference (AFD) values observed for pairwise comparisons of sampling locations ranged from 0.014 to 0.016 and pairwise genetics differentiation analyses indicated low levels of genetic differentiation among the three different sampling areas (Table 2). The inbreeding coefficient was 0.0609. The visualization of broad-scale population structure using a DAPC with all loci did not resolve any structure among individuals from CSL-MEX, GAL-ECU, and STR-ECU (Fig 2). Similarly, no divergence between sampling sites and no variation within sites was observed in the PCA (Fig 2). This lack of separation of individuals from the three sampling areas based on their genetic makeup is particularly evident when the individual density distribution of the first retained principal components from the discriminant function are examined (Fig 2).

**Table 2:** Pairwise genetic differentiation analyses for the yellowfin tuna among sampling areas.

		CSL-MEX	GAL-ECU	STR-ECU
***F*_*st*_**	CSL-MEX (n = 35)	-	0.00006	0.00001
	GAL-ECU (n = 88)	0.0004	-	0.00001
	STR-ECU (n = 41)	0.0005	0.0003	-

Sample sizes per sampling area are given in parentheses after the sampling area name; values above the diagonal are p-values; values below are F_*st*_-values.

Stock clustering analyses or structure are largely consistent with those of DAPC and PCA, showing a lack of structure among individuals in each group, and any differences between groups are minimal (S4 Fig). All Structure Selector analyses suggest a single cluster (S3 Fig). Similarly, the absence of groups were observed in the Clumpak cluster analyses, suggesting no structure in the population (S4 Fig).

## Discussion

A lack of genetic structure was observed for yellowfin tuna among the three sampling areas and no genetic difference by isolation was observed across spatial distances of 1,000–4,000 km, suggesting that yellowfin tuna within the EPO should currently be managed as a single stock. Pairwise analyses identified lowest genetic differences among individuals sampled at the two closest sampling sites, the Galápagos Islands and Santa Elena, Ecuador, separated by approximately 1,050 km. However, the greatest genetic differentiation was not observed between the two most distal locations (4,372 km) as might be expected if migratory distances played a role in the species differentiation, but rather among individuals from southern México and the Galápagos Islands (3,223 km). Structure analyses support the limited genetic differentiation observed among individuals from the different locations; low F_*ST*_ scores and AFD values suggest high levels of genetic homogeneity among yellowfin tuna from the three sampling sites. This is further supported by the Mantel test analysis and the low inbreeding coefficient. The absence of deficit or excess of heterozygotes allowed us to discard the Wahlund effect in the population [53]. The lack of observed genetic differentiation in yellowfin tuna populations within the EPO region, conflicts with a previous study using microsatellite markers [11]. Our findings suggest there is insufficient genetic population structure in this region of the EPO to support multiple yellowfin tuna stocks, and therefore yellowfin tuna within the EPO should be managed as a single transboundary fishery, in accordance with the IATTC. However, future studies that increase the sample size, such as more sampling locations, or utilize more advance genomic techniques (e.g., whole genome sequencing and close kin mark recapture) may more clearly define the limited population structure observed in this study and may challenge these findings.

Our results showed a similar allelic richness among the three locations. This result and the similar patterns found in the PCA of each region, support the idea of limited differentiation among the sampling areas. Nevertheless, allelic richness is only one measure of genetic diversity at a given time [54], so continuous monitoring is crucial for achieving good management plans. In 1961, Orange [55] described several areas of the Pacific Ocean near Central America, including the Revillagigedo archipelago in México, and Cocos Island in Costa Rica, as possible spawning grounds for yellowfin tuna. Recent observations reported within the past ten years also support these findings and have demonstrated high occurrences of yellowfin tuna larvae within this regional area of the EPO [56, 57]. These studies provide evidence for the limited genetic differentiation that was observed in yellowfin tuna across our sampling areas, despite being more than 2,000 km apart. According to a recent study, movements/migration patterns of the yellowfin tuna occurred between close areas and showed higher dispersion rates in the fish released in offshore areas compared to the individuals released close to the coast or around islands [58]. If the region around Cocos Island is a spawning area, native individuals could disperse both to northern and southern areas of the EPO, reaching all three sampling areas of this study. While there is potential for temporally displaced spawning aggregations to produce mixed stocks of yellowfin tuna in the EPO, additional spatially and temporally structured sampling programs will be required to further address this hypothesis.

In addition to overfishing and anthropogenic impacts (e.g. pollution, habitat disruption), tuna populations in the EPO face other important threats like oxygen-minimum zones, which occur at elevated frequencies in this region [59], and have expanded in size over the past 20 years [60]. Likewise, the rise of water temperatures, as a consequence of anthropogenically driven climate change, could also reduce the availability of oxygen for marine organisms like tuna [61]. Due to their high oxygen demand, low dissolved oxygen might be a limiting factor for tunas, restricting their movements to shallow waters [62]. Moreover, the historical warming of the ocean has had a negative effect on marine fisheries production [63] causing changes in species regional distributions and, therefore, affecting the long-term availability of the fisheries [64]. These alterations could influence the migration patterns of yellowfin tuna, disrupting the gene flow, which ultimately could lead to the fragmentation of the population [65–67]. It will be important to track any changes in genetic structure of yellowfin tuna in the EPO, and other commercial fisheries, as adaptive management related to these changes may be required. The limited genetic structure observed in this fishery should be considered as a metric or indicator within fisheries management policies in this area, especially considering that this population is declining [2].

Several countries in the region, especially Ecuador and México, profit from the intense fishing of this stock and, therefore, regional management and conservation strategies are required for this shared resource. Hillborn et al. [68] provide evidence that intense management measures generally result in stock recovering or, at least, staying close to management target levels. Management measures include: adopting total allowable catch limits, establishing seasonal closures, and, setting catch limits for the longline fishery and effort limits for the purse seine fishery [69]. Identify management actions that have worked in other fisheries, particularly tuna fisheries, and the harmonization of fishing regulations for yellowfin tuna throughout the EPO region could promote the sustainability of the fishery. In 2017, IATTC [70] established several management measures that included limiting the number of active fish aggregation devices (FADs) per vessel, a 72-day closure for purse seine vessels greater than 182 tons carrying capacity, and a seasonal closure of the purse seine fishery in “El Corralito”, towards west of the Galápagos Islands, where three species of tunas (yellowfin tuna, bigeye, and skipjack) converge. These measures were planned to be applied between 2022 and 2024 and their overall effect is yet to be evaluated [71]. Our results can be used as steppingstone for the design and implementation of national and regional management policies rigorously complied by all members of IATTC, which should be linked to conservation efforts like the recent expansion of the Galápagos Marine Reserve and the establishment of the Galápagos-Cocos Swimway, a marine corridor that protect species as they migrate between protected areas. Boerder et al. [72] implied that the Galápagos Marine Reserve has a net positive effect on pelagic fisheries associated to the archipelago, emphasizing the importance of large-scale marine protected areas as both fisheries management and biodiversity conservation tools [73]. Additionally, Orange [55] suggested that the area around the Galápagos Islands is a spawning area for yellowfin tuna, which should be investigated further. It is critical to continue establishing management measures focused on the Galápagos Islands, given their importance for biodiversity and the fisheries, using the most accurate data to avoid the fragmentation of yellowfin tuna populations.

To inform adaptive management, it is essential to perform NGS population studies of the yellowfin tuna fishery, in combination with ecological and reproductive biology studies. Moreover, it is important to design management policies that do not only focus on controlling fishing activity, but also manage other threats, such as climate change, illegal and unrecorded catches, and pollution, as part of an ecosystem-based management approach. Despite the existence of international tuna regulation agencies, it is critical that governments and local entities that promote the research collaborate in the development of common regulations that guarantee the survival of this species. Understanding key aspects of the status of the species (e.g. habitat range, abundance, and number of stocks) is critical for stakeholders to develop and establish appropriate management measures that promote the biological sustainability of the stocks, which ultimately guarantees the survival of the species and food security in the region [68].

## Supporting information

S1 Table SNP filtering summary using radiator (Gosselin et al. 2020) detailing the thresholds for each filter parameter. (TIF)

S1 Fig Genome-wide individual’s mean observed heterozygosity. (TIF)

S2 Fig Analysis of molecular variance (AMOVA) of yellowfin tuna populations amongst basins nested within sampling areas. (TIF)

S3 Fig Structure selector analyses. (PDF)

S4 Fig Clumpak cluster analyses. (PDF)

S1 File Metadata explanation of the SNPs single row file. (XLSX)

S2 File SNPs single row file. (RAR)

## References

[1] Burgess MG, Polasky S, Tilman D. Predicting over fi shing and extinction threats in multispecies fisheries. PNAS. 2013;110(40). 10.1073/pnas.1314472110 PMC3791778 24043810

[2] Collette B.B., Boustany A., Fox W., Graves J., uan Jorda M& R. Thunnus albacares (Yellowfin Tuna). The IUCN Red List of Threatened Species. 2021.

[3] FAO. The State of World Fisheries and Aquaculture 2020. The State of World Fisheries and Aquaculture 2020. 2020.

[4] Pons M, Branch TA, Melnychuk MC, Jensen OP, Brodziak J, Fromentin JM, et al. Effects of biological, economic and management factors on tuna and billfish stock status. Fish Fish. 2016;1–21.

[5] Pecoraro C, Babbucci M, Villamor A, Franch R, Papetti C, Leroy B, et al. Methodological assessment of 2b-RAD genotyping technique for population structure inferences in yellowfin tuna (Thunnus albacares). Mar Genomics. 2016;25:43–8. doi: 10.1016/j.margen.2015.12.002 26711352

[6] The Global Tuna. Developing management advice to rebuild the Indian Ocean yellowfin tuna (Thunnus albacares) stock in two generations. 2020.

[7] Grewe P, Feutry P, Hill P, Gunasekera R, Schaefer K, Itano D, et al. Evidence of discrete yellowfin tuna (Thunnus albacares) populations demands rethink of management for this globally important resource. Sci Rep. 2015;5:1–9. doi: 10.1038/srep16916 26593698 PMC4655351

[8] Barth J, Damerau M, Matschiner M, Jentoft S, Hanel R. Genomic Differentiation and Demographic Histories of Atlantic and Indo-Pacific Yellowfin Tuna (Thunnus albacares) Populations. Genome Biol Evol. 2017;9:1084–98. doi: 10.1093/gbe/evx067 28419285 PMC5408087

[9] ISSF. Status of the world fisheries for tuna: March 2019. Technical Report 2019–07. International Seafood Sustainability Foundation. Washington; 2019.

[10] IATTC. Status of the tuna and billfish stocks in 2018. Global Institutions and Social Knowledge. La Jolla, California (USA); 2019.

[11] Díaz P, Uribe M. Spatial differentiation in the eastern Pacific yellowfin tuna revealed by microsatellite variation. Fish Sci. 2006;72:590–6.

[12] Waples RS. Separating the Wheat From the Chaff: Patterns of Genetic Differentiation in High Gene Flow Species. 1998;438–50.

[13] Ely B, Viñas J, Alvarado J, Black D, Lucas L, Covello K, et al. Consequences of the historical demography on the global population structure of two highly migratory cosmopolitan marine fishes: the yellowfin tuna (Thunnus albacares) and the skipjack tuna (Katsuwonus pelamis). Evol Biol. 2005;5:1–9. 10.1186/1471-2148-5-19 PMC554763 15725349

[14] Pecoraro C, Babbucci M, Franch R, Rico C, Papetti C, Chassot E, et al. The population genomics of yellowfin tuna (Thunnus albacares) at global geographic scale challenges current stock delineation. Sci Rep. 2018;8(1):1–10. 30224658 10.1038/s41598-018-32331-3 PMC6141456

[15] Comisión Interamericana del Atún Tropical. Informes de captura, datos y otros informes [Internet]. Informes de captura, datos y otros informes. 2019. Available from: https://www.iattc.org/HomeSPN.htm

[16] Sibert J, Hampton J. Mobility of tropical tunas and the implications for fisheries management. Mar Policy. 2003;27:87–95.

[17] Graham B, Koch P, Newsome S, McMahon K, Aurioles D. Using Isoscapes to Trace the Movements and Foraging Behavior of Top Predators in Oceanic Ecosystems. In: West J, Bowen G, Dawson T, Tu K, editors. Isoscapes: Understanding Movement, Pattern, and Process on Earth Through Isotope Mapping. Springer Netherlands; 2010. p. 299–318.

[18] Minte-Vera C, Maunder M, Aires-da-Silva A. Status of yellowfin tuna in the Eastern Pacific Ocean in 2017 and outlook for the future. Scientific Advisory Commitee. La Jolla, California; 2017.

[19] Schaefer K, Fuller D, Aldana G. Movements, behavior, and habitat utilization of yellowfin tuna (Thunnus albacares) in waters surrounding the Revillagigedo Islands Archipelago Biosphere Reserve, Mexico. Fish Oceanogr. 2014;23:65–82.

[20] Hoolihan J, Wells D, Luo J, Falterman B, Prince E, Rooker J. Vertical and Horizontal Movements of Yellowfin Tuna in the Gulf of Mexico. Mar Coast Fish Dyn Manag Ecosyst Sci. 2014;6:211–22.

[21] Schaefer K, Fuller D. Horizontal movements, utilization distributions, and mixing rates of yellowfin tuna (Thunnus albacares) tagged and released with archival tags in six discrete areas of the eastern and central Pacific Ocean. Fish Oceanogr. 2022;31(August 2021):84–107.

[22] Moore BR, Lestari P, Cutmore SC, Proctor C, Lester RJG. Article Movement of juvenile tuna deduced from parasite data. ICES J Mar Sci. 2019;76(6):1678–89.

[23] Logan JM, Pethybridge H, Lorrain A, Somes CJ, Allain V, Bodin N, et al. Global patterns and inferences of tuna movements and trophodynamics from stable isotope analysis. Deep Res Part II Top Stud Oceanogr. 2020;175(March).

[24] Lam CH, Tam C, Kobayashi DR, Lutcavage ME. Complex Dispersal of Adult Yellowfin Tuna From the Main Hawaiian Islands. 2020;7(March):1–13.

[25] Wright SR, Righton D, Naulaerts J, Schallert RJ, Bendall V, Griffiths C, et al. Fidelity of yellowfin tuna to seamount and island foraging grounds in the central South Atlantic Ocean. Deep Res Part I. 2021;172:103513.

[26] Anderson G, Holland E, Rico C. Meta-analysis of Factors Influencing Population Differentiation in Yellowfin Tuna (Thunnus albacares). Rev Fish Sci Aquac [Internet]. 2019;27(4):517–32. Available from: 10.1080/23308249.2019.1633270

[27] Butler JM. Single Nucleotide Polymorphisms and Applications. In: Elservier, editor. Advanced Topics in Forensic DNA Typing. 2012. p. 347–69.

[28] Ozerov M, Vasemagi A, Wennevik V, Diaz-fernandez R, Kent M, Gilbey J, et al. Finding Markers That Make a Difference: DNA Pooling and SNP-Arrays Identify Population Informative Markers for Genetic Stock Identification. PLOSONE. 2013;8(12). doi: 10.1371/journal.pone.0082434 24358184 PMC3864958

[29] Moore BR, Bell JD, Evans K, Farley J, Grewe PM, Hampton J, et al. Defining the stock structures of key commercial tunas in the Pacific Ocean I: Current knowledge and main uncertainties. Fish Res [Internet]. 2020;230(May 2019):105525. Available from: 10.1016/j.fishres.2020.105525

[30] Hauser L, Carvalho GR. Paradigm shifts in marine fisheries genetics: ugly hypotheses slain by beautiful facts. Fish Fish. 2008;9:333–62.

[31] Laconcha U, Iriondo M, Arrizabalaga H, Manzano C, Markaide P, Montes I, et al. New Nuclear SNP Markers Unravel the Genetic Structure and Effective Population Size of Albacore Tuna (Thunnus alalunga). PLOSONE. 2015;10(6):1–19. 10.1371/journal.pone.0128247 PMC4474438 26090851

[32] Bourlat SJ, Borja A, Gilbert J, Taylor MI, Davies N, Weisberg SB, et al. Genomics in marine monitoring: New opportunities for assessing marine health status. Mar Pollut Bull [Internet]. 2013;74(1):19–31. Available from: doi: 10.1016/j.marpolbul.2013.05.042 23806673

[33] Reid NM, Proestou DA, Clark BW, Warren WC, Colbourne JK, Shaw JR, et al. The genomic landscape of rapid repeated evolutionary adaptation to toxic pollution in wild fish. Science (80-). 2016;354(6317):1305–8. doi: 10.1126/science.aah4993 27940876 PMC5206662

[34] Bernatchez L, Wellenreuther M, Araneda C, Ashton DT, Barth JMI, Beacham TD, et al. Harnessing the Power of Genomics to Secure the Future of Seafood. Trends Ecol Evol [Internet]. 2017;32:665–80. Available from: doi: 10.1016/j.tree.2017.06.010 28818341

[35] Mullins RB, Mckeown NJ, Sauer WHH, Shaw PW. Genomic analysis reveals multiple mismatches between biological and management units in yellowfin tuna (Thunnus albacares). Int Counc Explor Sea J Mar Sci. 2018.

[36] Foster SD, Feutry P, Grewe P, Davies C. Sample size requirements for genetic studies on yellowfin tuna. PLoS One [Internet]. 2021;16(11 November):1–17. Available from: doi: 10.1371/journal.pone.0259113 34735482 PMC8568148

[37] Anderson G, Lal M, Hampton J, Smith N, Rico C. Close Kin Proximity in Yellowfin Tuna (Thunnus albacares) as a Driver of Population Genetic Structure in the Tropical Western and Central Pacific Ocean. Front Mar Sci [Internet]. 2019;6(July):1–15. Available from: https://www.frontiersin.org/article/10.3389/fmars.2019.00341/full

[38] Anderson G, Lal M, Stockwell B, Hampton J, Smith N, Nicol S, et al. No Population Genetic Structure of Skipjack Tuna (Katsuwonus pelamis) in the Tropical Western and Central Pacific Assessed Using Single Nucleotide Polymorphisms. Front Mar Sci. 2020;7:1–17.32802822

[39] Sansaloni C, Franco J, Santos B, Percival-Alwyn L, Singh S, Petroli C, et al. Diversity analysis of 80,000 wheat accessions reveals consequences and opportunities of selection footprints. Nat Commun [Internet]. 2020;11(1):1–12. Available from: 10.1038/s41467-020-18404-w 32917907 PMC7486412

[40] Gosselin Thierry, Lamothe Manuel, Floriaan Devloo-Delv PG. Radiator: RADseq Data Exploration, Manipulation and Visualization using R. [Internet]. 2020. Available from: https://thierrygosselin.github.io/radiator/

[41] RStudio Team. RStudio: Integrated Development for R. [Internet]. Boston, MA; 2020. Available from: http://www.rstudio.com/

[42] Jombart T AI. adegenet 1.3–1: new tools for the analysis of genome-wide SNP data. Bioinformatics. 2011;1403–5. doi: 10.1093/bioinformatics/btr521 21926124 PMC3198581

[43] Zhian N., Kamvar Javier F.,Jonah C. Brooks, Stacy A. Krueger-Hadfield, Erik Sotka, Brian J. Knaus, Patrick G. Meirmans FDC, Grünwald NJ. poppr. 2020.

[44] Gruber B, Unmack PJ, Berry OF, Georges A. dartr: An r package to facilitate analysis of SNP data generated from reduced representation genome sequencing. Mol Ecol Resour. 2018;691–699. doi: 10.1111/1755-0998.12745 29266847

[45] Stoffel MA, Esser M, Kardos M, Humble E, Nichols H, David P, et al. inbreedR: an R package for the analysis of inbreeding based on genetic markers. Methods Ecol Evol. 2016;7(11):1331–9.

[46] Endelman JB. Ridge Regression and Other Kernels for Genomic Selection with R Package rrBLUP. Plant Genome. 2011;4(November):250–5.

[47] Berner D. Allele frequency difference AFD-an intuitive alternative to FST for quantifying genetic population differentiation. Genes (Basel). 2019;10(4). 10.3390/genes10040308 PMC6523497 31003563

[48] Pritchard J, Stephens M, Donnelly P. Inference of Population Structure Using Multilocus Genotype Data. Genetics. 2000;155:945–59. doi: 10.1093/genetics/155.2.945 10835412 PMC1461096

[49] Jakobsson M, Rosenberg N. CLUMPP: a cluster matching and permutation program for dealing with label switching and multimodality in analysis of population structure. Bioinformatics. 2007;23:1801–6. doi: 10.1093/bioinformatics/btm233 17485429

[50] Archer FI, Adams PE, Schneiders BB. stratag: An r package for manipulating, summarizing and analysing population genetic data. Mol Ecol Resour. 2016;17(1):5–11. doi: 10.1111/1755-0998.12559 27327208

[51] long Li Y, xian Liu J. STRUCTURE SELECTOR: A web-based software to select and visualize the optimal number of clusters using multiple methods. Mol Ecol Resour. 2018;18:176–7. doi: 10.1111/1755-0998.12719 28921901

[52] Kopelman NM, Mayzel J, Jakobsson M, Rosenberg NA, Mayrose I. CLUMPAK: a program for identifying clustering modes and packaging population structure inferences across K. Mol Ecol Resour. 2015;5:1179–91. doi: 10.1111/1755-0998.12387 25684545 PMC4534335

[53] Garnier‐Géré P, Chikhi L. Population Subdivision, Hardy–Weinberg Equilibrium and the Wahlund Effect. eLS. 2013;1–5.

[54] Padrón M, Guizien K. Modelling the effect of demographic traits and connectivity on the genetic structuration of marine metapopulations of sedentary benthic invertebrates. ICES J Mar Sci. 2016;73(7):1935–45.

[55] Orange CJ. Spawning of yellowfin tuna and skipjack in the eastern tropical Pacific, as inferred from studies of gonad development. Bull Inter-American Trop Tuna Comm IATTC. 1961;5(6):459–526.

[56] Reglero P, Tittensor DP, Álvarez-Berastegui D, Aparicio-González A, Worm B. Worldwide distributions of tuna larvae: Revisiting hypotheses on environmental requirements for spawning habitats. Mar Ecol Prog Ser. 2014;501:207–24.

[57] Schaefer KM, Fuller DW. Spatiotemporal variability in the reproductive biology of yellowfin tuna (Thunnus albacares) in the eastern Pacific Ocean. Fish Res [Internet]. 2022;248(October 2021):106225. Available from: 10.1016/j.fishres.2022.106225

[58] Schaefer KM, Fuller DW. Horizontal movements, utilization distributions, and mixing rates of yellowfin tuna (Thunnus albacares) tagged and released with archival tags in six discrete areas of the eastern and central Pacific Ocean. Fish Oceanogr. 2022;31(1):84–107.

[59] Karstensen J, Stramma L, Visbeck M. Oxygen minimum zones in the eastern tropical Atlantic and Pacific oceans. Prog Oceanogr. 2008;77(4):331–50.

[60] Lam VWY, Allison EH, Bell JD, Blythe J, Cheung WWL, Frölicher TL, et al. Climate change, tropical fisheries and prospects for sustainable development. Nat Rev Earth Environ. 2020.

[61] Oschlies A, Brandt P, Stramma L, Schmidtko S. Drivers and mechanisms of ocean deoxygenation. Nat Geosci [Internet]. 2018;11(7):467–73. Available from: 10.1038/s41561-018-0152-2

[62] Nimit K, Masuluri NK, Berger AM, Bright RP, Prakash S, TVS U, et al. Oceanographic preferences of yellowfin tuna (Thunnus albacares) in warm stratified oceans: A remote sensing approach. Int J Remote Sens [Internet]. 2020;41(15):5785–805. Available from: 10.1080/01431161.2019.1707903

[63] Free CM, Thorson JT, Pinsky ML, Oken KL, Wiedenmann J, Jensen OP. Impacts of historical warming on marine fisheries production. Science (80-). 2019;363(6430):979–83. 10.1126/science.aau1758 30819962

[64] Wu CC, Chiang H, Chou Y, Wong Z, Hsu C, Chen C, et al. Phylogeography of yellowfin tuna (Thunnus albacares) in the Western Pacific and the Western Indian Oceans inferred from mitochondrial DNA. Fish Res. 2010;105:248–53.

[65] Halpern Benjamin S., Walbridge Shaun, Selkoe Kimberly A., Kappel Carrie V., Micheli Fiorenza, 3 Caterina D’Agrosa, et al. A Global Map of Human Impact on Marine Ecosystems. Science (80-). 2008;319(February):948–53. 10.1126/science.1149345 18276889

[66] Liu J, Mooney H, Hull V, Davis SJ, Gaskell J, Hertel T, et al. Systems integration for global sustainability. Science (80-). 2015;347(6225). 10.1126/science.1258832 25722418

[67] Ortuño G, Dunn D. Review Article A review of the impacts of fisheries on open-ocean ecosystems. Int Counc Explor Sea J Mar Sci. 2017.

[68] Hilborn R, Oscar R, Anderson CM, Baum JK, Branch TA, Costello C, et al. Effective fisheries management instrumental in improving fish stock status. PNAS. 2020;117(4):2218–24. doi: 10.1073/pnas.1909726116 31932439 PMC6995010

[69] Post V, Squires D. Managing Bigeye Tuna in the Western and Central Pacific Ocean. Front Mar Sci. 2020;7(August):1–8.32802822

[70] IATTC. Resolution C-17-02 Conservation Measures for Tropical Tunas in the Eastern Pacific Ocean During 2018–2020 and Amendment To Resolution C-17-01. 2017;(July 2017):3–7.

[71] Commission IATT. Inter-American Tropical Tuna Commission 90 Th Meeting Minutes of the Meeting. 2021.

[72] Boerder K, Bryndum-Buchholz A, Worm B. Interactions of tuna fisheries with the Galápagos marine reserve. Mar Ecol Prog Ser. 2017;585(December):1–15.

[73] Relano V, Pauly D. Philopatry as a Tool to Define Tentative Closed Migration Cycles and Conservation Areas for Large Pelagic Fishes in the Pacific. Sustainability. 2022;14(5577).

