# Correction: Lack of genetic differentiation in yellowfin tuna has conservation implications in the Eastern Pacific Ocean

**DOI:** 10.1371/journal.pone.0345206

**Published:** 2026-03-16

**Authors:** The PLOS One Staff

The Data Availability statement for this article is incorrect. The correct statement is: All data is available from https://doi.org/10.5061/dryad.qrfj6q5wj.

The publisher apologizes for the error.
