# Supplementary figures and images for: Lack of genetic differentiation in yellowfin tuna has conservation implications in the Eastern Pacific Ocean
Source: PLoS One. 2022 Aug 30;17(8):e0272713. doi: 10.1371/journal.pone.0272713 (PMC9426925; doi:10.1371/journal.pone.0272713)

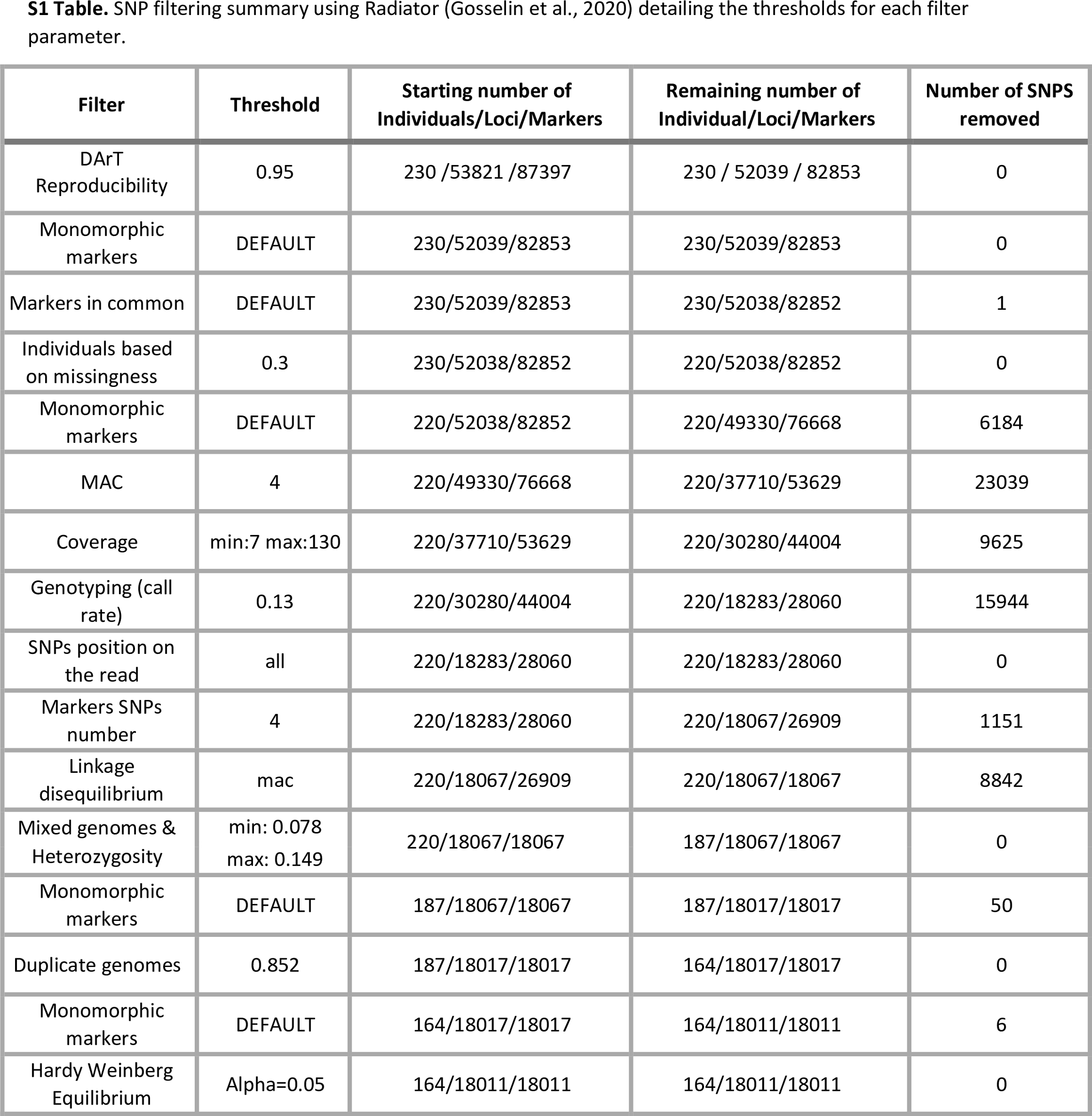

Supplement: S1 Table — (TIF) [file pone.0272713.s001.tif]

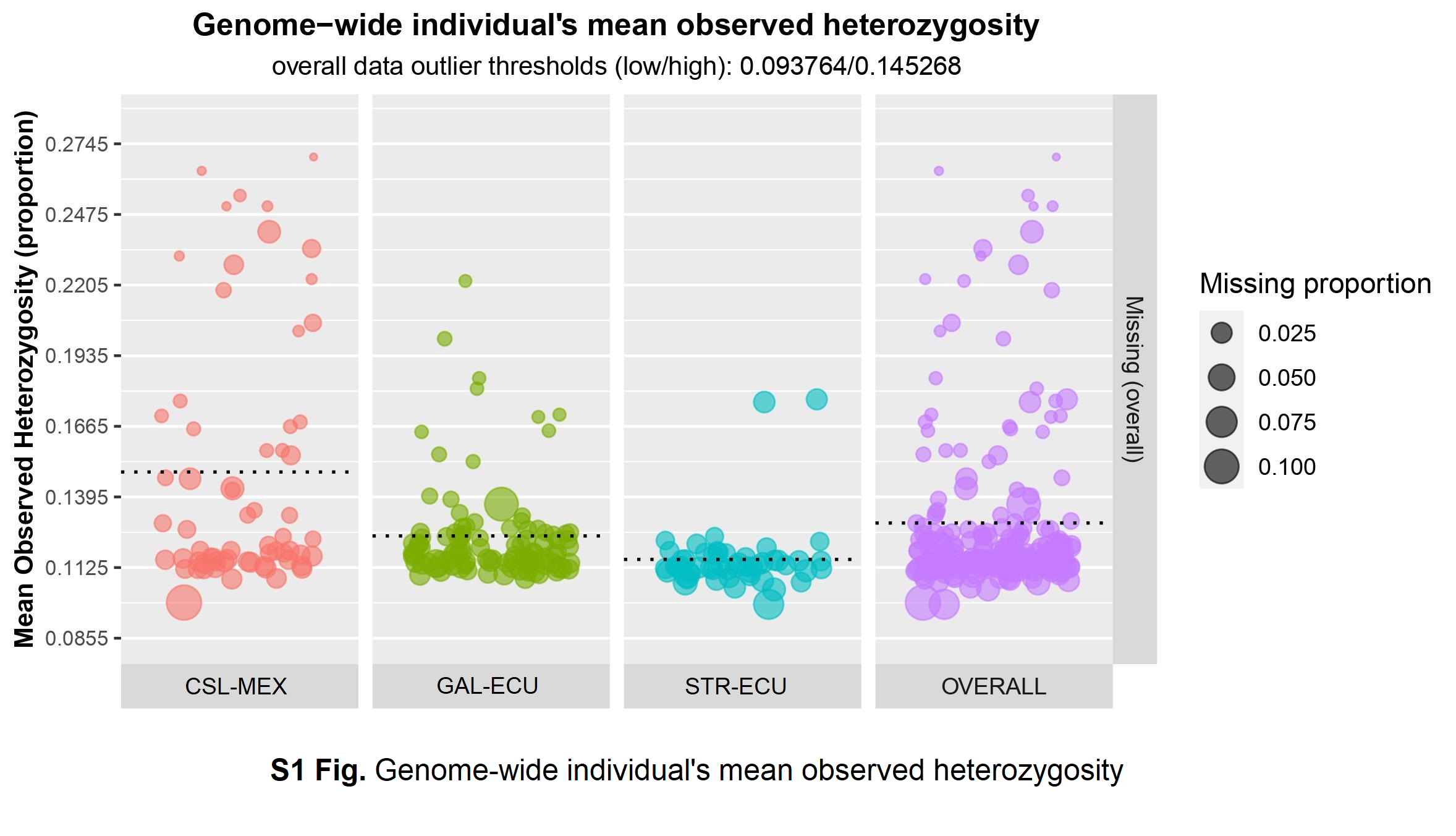

Supplement: S1 Fig — (TIF) [file pone.0272713.s002.tif]

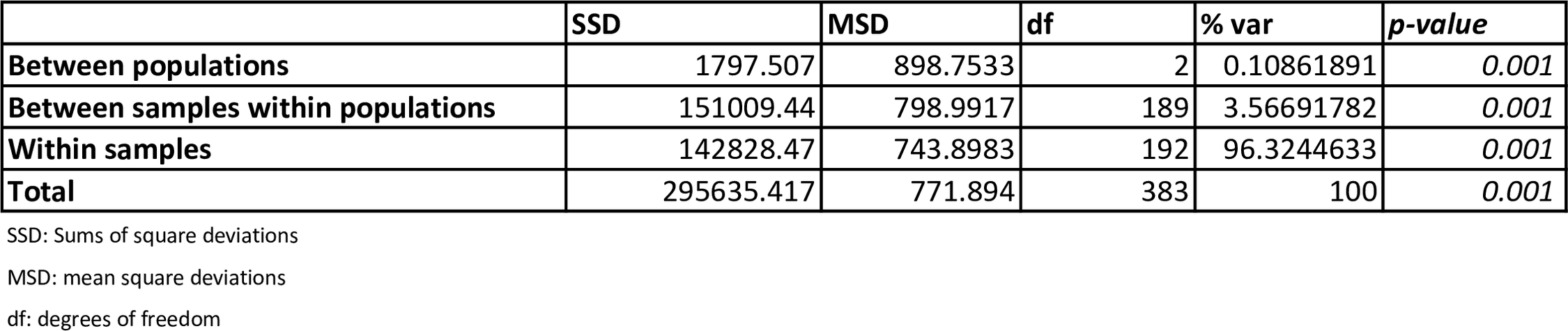

Supplement: S2 Fig — (TIF) [file pone.0272713.s003.tif]

**MedMed K**

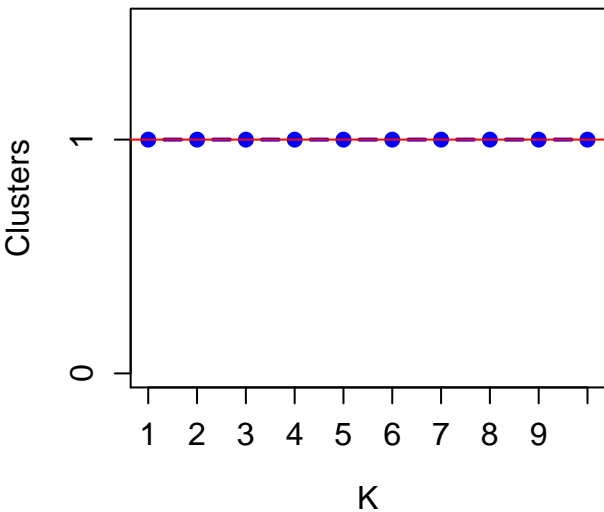

**MedMean K**

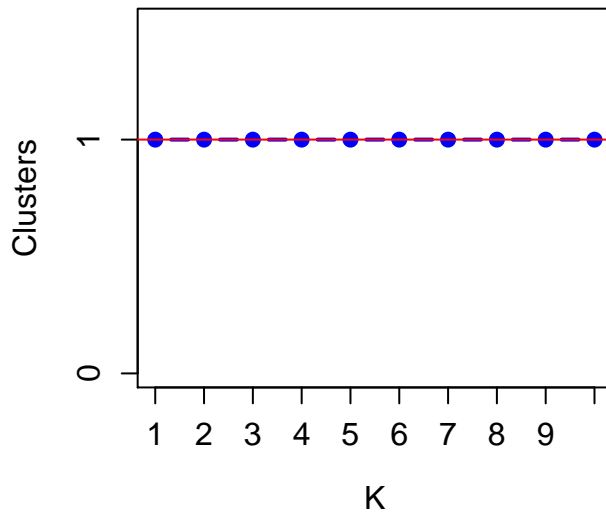

**MaxMed K**

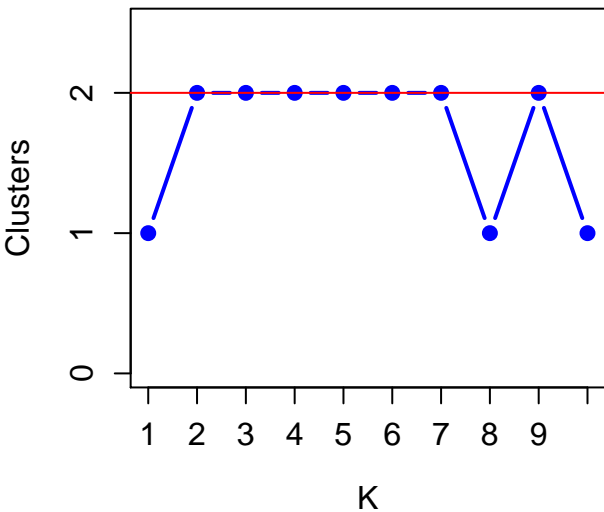

**MaxMean K**

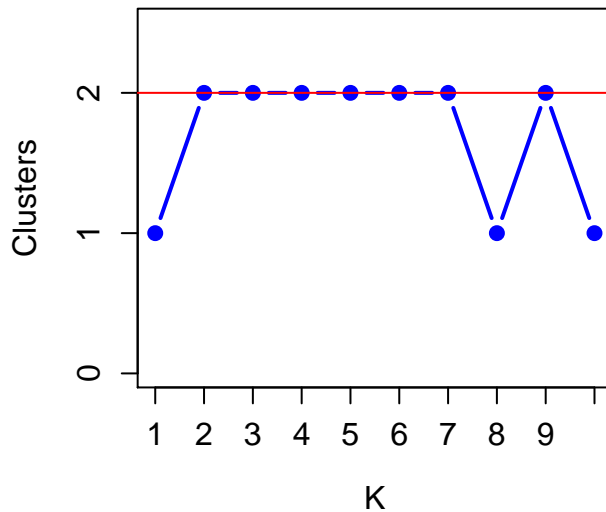

Supplement: S3 Fig — (PDF) [file pone.0272713.s004.pdf]
