# Supplementary material for: Lack of genetic differentiation in yellowfin tuna has conservation implications in the Eastern Pacific Ocean
Source: PLoS One. 2022 Aug 30;17(8):e0272713. doi: 10.1371/journal.pone.0272713 (PMC9426925; doi:10.1371/journal.pone.0272713)
Supplement: S4 Fig — (PDF) [file pone.0272713.s005.pdf]

CLUMPAK main pipeline - Job 1653093633 summary

Major modes for the uploaded data:

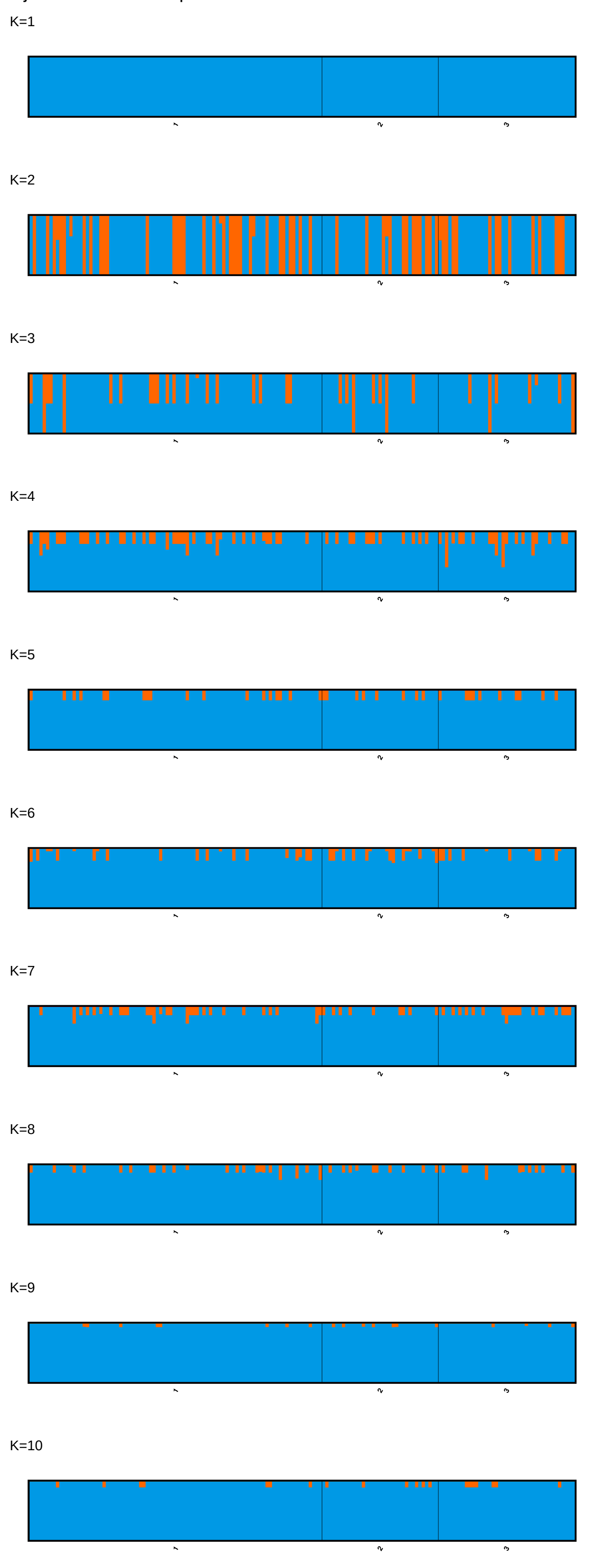

Minor modes for the uploaded data:

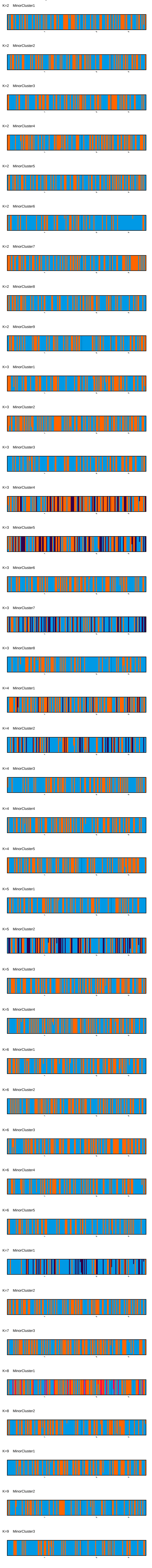

Division of runs by mode:

K=1	10/10
K=2	1/10, 1/10, 1/10, 1/10, 1/10, 1/10, 1/10, 1/10, 1/10, 1/10
K=3	2/10, 1/10, 1/10, 1/10, 1/10, 1/10, 1/10, 1/10, 1/10
K=4	5/10, 1/10, 1/10, 1/10, 1/10, 1/10
K=5	6/10, 1/10, 1/10, 1/10, 1/10
K=6	5/10, 1/10, 1/10, 1/10, 1/10, 1/10
K=7	7/10, 1/10, 1/10, 1/10
K=8	8/10, 1/10, 1/10
K=9	6/10, 1/10, 1/10, 1/10, 1/10
K=10	10/10
